# Persistence at the final stage of volcanic island ontogeny: Abiotic predictors explain native plant species richness on 111 remote Pacific atolls

**DOI:** 10.1002/ece3.4680

**Published:** 2018-11-20

**Authors:** Sébastien Larrue, Jean‐François Butaud, Curtis C. Daehler, Stéphane Ballet, Julien Chadeyron, Roger Oyono

**Affiliations:** ^1^ University Clermont Auvergne (UCA), GEOLAB‐CNRS Clermont‐Ferrand France; ^2^ Consultant in Forestry and Polynesian Botany Papeete French Polynesia; ^3^ Department of Botany University of Hawaii at Manoa Honolulu Hawaii; ^4^ Aix‐Marseille Université, CNRS, Centrale Marseille Marseille France; ^5^ Department of Geography University Clermont Auvergne (UCA) Clermont‐Ferrand France; ^6^ University of French Polynesia, GAATI Faa’a French Polynesia

**Keywords:** coral atolls, general dynamic model, human impact, island biogeography, native species richness pattern, Pacific Ocean, spatial and physical predictors

## Abstract

**Aim:**

The final island ontogeny of the general dynamic model (GDM) (i.e., before island submergence) in tropical oceans corresponds to the coral atoll stage. Here, we examined whether the species richness of native vascular plants (indigenous and endemic species) on atolls is controlled by spatial and/or physical processes. We also predicted that atolls strongly affected by anthropogenic disturbance would have lower native species richness than predicted by spatial and physical processes.

**Location:**

Marshall Islands, Kiribati Islands, Nauru, Niue, Johnston, Cook Islands, French Polynesia and Pitcairn Islands (Pacific Ocean).

**Taxon:**

Native vascular plants.

**Methods:**

We used stepwise regression to test the relative influence of five biogeographic variables on native species richness. Relationships were assessed for the full set of 111 Pacific coral atolls, as well as for atoll subsets ranging from 9 to 45 atolls. An index of human impact was then estimated, and residuals in the regression model predicting species richness from biogeographic variables were compared with the level of human impact.

**Results:**

A regression model including atoll area, highest atoll elevation, the stepping stone distances from the nearest raised atoll and volcanic island explained native species richness on the 111 Pacific coral atolls. Regression models for different archipelagos and atoll subsets were also significant. Endemic species richness was significantly linked with highest atoll elevation and the stepping stone distances from the nearest raised atoll. Residuals in the biogeographic regression model were barely related to human impact across the 111 atolls but were significantly related to human impact in the Kiribati atolls.

**Main conclusions:**

Native species richness on atolls is mainly controlled by physical and spatial characteristics. However, anthropogenic disturbances have altered the predicted pattern of native species richness leading to a lower model fit in some atoll subsets.

## 1 INTRODUCTION

Island biogeographers have developed various models and theories to explain native diversity on islands (for review see Lomolino, 2016). The general dynamic model (GDM) is the most recent theory in island biogeography (Borregaard, Matthews, & Whittaker, 2016; Otto et al., 2016; Steinbauer, Klara, Field, Reineking, & Beierkuhnlein, 2013; Whittaker et al., 2007; Whittaker, Triantis, & Ladle, 2008, 2010 ). The GDM gathers geomorphological and biological processes within an evolutionary timescale. The main precept of the GDM is that the ontogeny of volcanic islands (i.e., emergence, maturity, subsidence, and island submergence) leads to predictable changes in island elevation, island area, and topographic heterogeneity, generating predictable changes in the rates of immigration, speciation, species extinction, and species richness.

**Figure 1:**
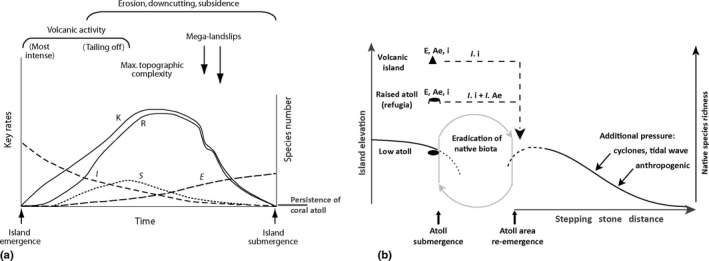
(a) The general dynamic theory GDM with extinction (E), immigration (I), carrying capacity (K), species richness (R), and speciation curve (S) (Whittaker, Triantis, & Ladle, 2008). Note that “persistence of coral atoll” has been added; (b) Hypothetical causal relationships between native species richness (black curve) and abiotic factors at the final volcanic island ontogeny herein formed by the persistence of coral atoll with: (E) single‐island endemic, (Ae) archipelago‐level endemic, (i) indigenous, and immigration (dashed line) of indigenous (*I*.i) and archipelago‐level endemic species (*I*.Ae). “Gray loop” represents eustatic sea‐level variations due to last glacial–interglacial cycle of Pleistocene period and Holocene highstand

In the tropical zone, the final volcanic island ontogeny (i.e., before island submergence, here after called “final stage”) corresponds closely to low coral atolls (Stuessy, 2007; Whittaker & Fernández‐Palacios, 2007). An atoll is a flat and low island with a ring‐shaped coral reef and limestone substrates composed of skeletons of coral and foraminifera established on the submerged flanks of an eroded volcanic island (Mueller‐Dombois, 2002). At this final stage, we hypothesized that native species richness of vascular plants will be driven mostly by a combination of simple spatial and abiotic predictors (Figure 1).

Coral atolls have attracted less interest in island biogeography than volcanic islands, due to difficult geographic access for researchers and a very low number of endemic species. Atolls often have homogeneous geomorphology, that is, similar landforms, with low elevation and no orographic rainfall, thus providing low habitat diversity (Manner, 1995; Mueller‐Dombois & Fosberg, 1998; Stoddart, 1992), and similar ages (Dickinson, 2004). Consequently, the atolls can be viewed as “simplified” islands (Thaman, 2008) with comparable environments, suitable for investigating native species richness patterns.

Formerly, some atoll characteristics have been related to native species richness (indigenous and endemic species). A species–area relationship (SAR) has been observed on atolls (Niering, 1963; Woodroffe, 1986) but other studies reported only a weak species–area relationship on low coral islands of the Pacific Ocean (Manner, 1995; Mueller‐Dombois & Fosberg, 1998; Stoddart, 1992). Fosberg (1976), Stoddart (1992); Woodroffe (2008) observed an increase in species richness with increasing atoll elevation, possibly related to submersion of the narrowest and lowest atolls during extreme weather events.

Additionally, the coral platform of atolls has evolved in response to a cycle of sea‐level variation during the Quaternary (Ohde et al., 2002). Changes in island area and submergence during eustatic sea‐level variation, due to glacial–interglacial cycles of the Pleistocene period and Holocene highstand, have more strongly impacted the native flora on low atolls relative to raised atolls and volcanic islands (Camoin, Ebren, Eisenhauer, Bard, & Faure, 2001; Dickinson, 2001; Hewitt, 2000; Mueller‐Dombois & Fosberg, 1998), suggesting regression or extirpation of island biota on the low islands (Cibois, Thibault, & Pasquet, 2010). Thus, the stepping stone distance to the nearest volcanic island or raised coral atoll (as refugia) could be an important factor explaining native species richness on atolls (Cibois et al., 2010; Larrue, Butaud, Dumas, & Ballet, 2015). However, it is well known that factors interact across space and time and significant predictors differ due to different scales of geographical analyses, the sample of islands and studied taxa, habitat diversity and degree of disturbance, geographic isolation, geological history, as well as stochastic events (e.g., Kalmar & Currie, 2006; Triantis et al., 2006; Triantis, Guilhaumon, & Whittaker, 2012; Triantis, Economo, Guilhaumon, & Ricklefs, 2015; Whittaker, Willis, & Field, 2001).

Here, five biogeographic variables were considered to explain native species richness (including a focus on endemic species richness) for 111 atolls in the Pacific Ocean. However, considering that oceanic island biodiversity may be impacted by human colonization and anthropogenic habitat conversion, contemporary native species richness patterns may be altered by humans (e.g., Gillespie, 2007), especially on atolls, which are among the most highly threatened and degraded oceanic islands (Thaman, 2008). To test this latter prediction, we estimated the level of human impact on each atoll, and predicted that atolls having lower than expected native richness (large negative residuals) in the stepwise regression model based on biogeographic variables would be atolls that have experienced more extreme anthropogenic impacts.

## 2 MATERIALS AND METHODS

### 2.1 Atolls surveyed

The atolls surveyed are located between 19°16'48.8''N–24°40'49.8''S and 166°38'53.7''E–124°47'16.9''W (meridian 180° in the area). The study area spans over a tropical region influenced by southeasterly or northeasterly Trade winds, where the mean annual sea‐level rainfall tends to decrease from west to east with ~4,000 mm around the Marshall Islands and 1,700 mm around the Pitcairn Islands (Mueller‐Dombois, 2002).

The 111 atolls sampled include atolls of French Polynesia (Austral, Society, Tuamotu, and Gambier) and Pitcairn (*n* = 45), Cook atolls (*n* = 9), Marshall atolls (*n* = 28), Kiribati atolls (*n* = 26) and the atolls of Nauru, Niue, and Johnston (*n* = 3). The atoll sample contained 106 low coral atolls and five raised atolls ≥20 m a.s.l.: Makatea (113 m a.s.l., Tuamotu, French Polynesia), Banaba (81 m a.s.l., Kiribati Islands), Niue (73 m a.s.l.), Nauru (71 m a.s.l.), and Henderson (33 m a.s.l., Pitcairn Islands). The raised coral atolls used in this study are composed of an elevated terrestrial plateau (i.e., old lagoon floor formed by coral conglomerate) surrounded by an eroded coral ring, both have been uplifted by lithospheric flexure caused by the crustal loading of neighboring volcanic islands (e.g., Spencer, 1989).

Radiocarbon analysis of the coral conglomerate platform has been made in some sampled low atolls, providing information about ages of the atoll surfaces. Ages of 4,000–3,000 year BP were estimated for the Cocos Islands (Australia) (Woodroffe, McLean, & Wallensky, 1994); similar ages were obtained in the Kiribati and Tuvalu atolls ranging from *c*. 4,000–2,000 year BP (McLean & Hosking, 1991) and 2,120 year BP on Enewetak Atoll (Marshall Islands) (Szabo, Tracey, & Goter, 1985). In the Tuamotu atolls (French Polynesia), radiocarbon ages ranged between *c*. 5,500–2,500 year BP (Pirazzoli & Montaggioni, 1988). The ages of Kiribati and Marshall atolls seem broadly comparable while some low Tuamotu atolls may be slightly older.

The coral conglomerate surfaces of the five raised atolls were older. The ages range from Mio‐Pliocene to Plio‐Pleistocene (Jacobson & Hill, 1980; Montaggioni et al., 1985; Pandolfi, 1995), that is, 5–0.3 Ma year. Thus, these raised atolls were uplifted before the last interglacial sea‐level (late Pleistocene, *c*. 125 ka BP) and highstand of Holocene sea‐level (*c*. 8 ka BP). The sampled atolls were never connected to a mainland, and they are among the most isolated islands in the world in terms of their distance from continental or major volcanic land masses (Mueller‐Dombois, 2002).

### 2.2 Native species richness on the atolls

Here, the number of indigenous (i.e., native but not endemic) and endemic vascular plants (single‐atoll endemics plus archipelago‐level endemics) were well documented by different sources such as botanical surveys and database. This point was the most important criteria to select the 111 atolls. Indigenous and endemic species were summed to obtain native species richness. Various botanical sources from 1953 to 2017 were consulted.

Species richness on the Marshall and Kiribati atolls was recorded mainly from Fosberg (1953, 1955, 1959, 1985, 1990), Fosberg and Sachet (1962, 1969, 1987), Fosberg and Stoddart (1994), Releford, Stevens, Bridges, and Mc Clatchey (2009), Sabath (1977), and Thaman and Samuelu (2016). An online biodiversity database was used for the Cook atolls (McCormack, 2007) whereas data for the Pitcairn atolls were extracted from additional sources (Florence, Waldren, & Chepstow Lusty, 1995; Kingston, 2001; St John & Philipson, 1960; Waldren, Florence, & Chepstow‐Lusty, 1995). For the atolls of French Polynesia, we used various reports (e.g., Stoddart & Sachet, 1969) with the online database Nadeaud (Florence, Chevillotte, Ollier, & Meyer, 2007) and botanical surveys (Butaud, 2011, 2013 ; Butaud & Jacq, 2017; Meyer, 2013; Taputuarai, 2015). Native species richness was updated by using the last data for each archipelago. Note that native species on the atolls are mostly formed by widely dispersed indigenous species and these species are commonly observed by botanists during field survey through time.

### 2.3 Characteristics of the coral atolls

We selected five biogeographic variables as potential predictors of native plant species richness on the 111 coral atolls: (a) coastline length (km); (b) atoll area (km^2^); (c) highest atoll elevation (m); (d) the distance from the nearest raised coral atoll ≥20 m a.s.l. (km) (as refugia), and (e) the distance from the nearest volcanic island ≥100 km^2^ (km) (as a stepping stone distance). Here, climatic conditions of atolls were not selected as variables mainly because (a) total annual rainfall per atoll experiences extreme interannual variability due to variations in the El Ninõ–Southern Oscillation (ENSO) phenomenon (Morrison & Woodroffe, 2009; Stoddart, 1992) and (b) many atolls have no field data for total precipitation.

Measurements of coastline and distances were performed with a Geographic Information System (GIS Mapinfo® Professional version 10, WGS 1984 projection). A raised atoll ≥20 m a.s.l. was chosen as the criteria for defining potential refugia because during the last interglacial period (*c*. 125 ka), sea‐level was estimated ~5–7 m above the present sea‐level in the islands (Dickinson, 2001, 2004 ) with other estimates in the range of 9–13 m (see Montaggioni et al., 1985). There is no doubt that during this highstand, raised atolls ≥20 m a.s.l. remained emerged and protected from cyclonic waves. Atoll area (i.e., surface of emerged land), and highest atoll elevation, were obtained from the Atlas of coral reefs in French Polynesia (Andréfouët, Chauvin, Spraggins, Torres‐Puzilla, & Kranenbourg, 2005) and United Nations Environment Programme (UNEP) island database (https://islands.unep.ch), although the highest elevation of some atolls was obtained from various other sources.

### 2.4 Level of human impact

We estimated the level of potential human impact by the sum of seven criteria including recent and past human pressures. First, we used “Human Threat Indicator” (0–3) and “Invasive Species Indicator” (here, 0–3) from Dahl (1998). We then completed the level of human impact by summed other historical criteria: presence of coconut monoculture (+1), military installations (+1), devastation during World War II (+1), nuclear testing (+1), and phosphate mining (+1). In order to avoid subjectively weighing of these additional criteria, we used only presence/absence codification (1/0). The summed score was then assessed as “Low impact ≤3”, “Medium impact 3–6”, and “High impact>6”.

### 2.5 Statistical analysis

Stepwise regression was used to observe the relationships between native species richness and the five biogeographic variables. Additionally, the level of human impact was also included among the variables.

We detected significant collinearity between “atoll area” and “coastline length” in the overall analysis of 111 atolls as well as archipelago subsets of atolls (not shown). So, only the best single fitting variable among “atoll area” and “coastline length” was included among the other biogeographic variables in stepwise models. We then used a stepwise regression procedure to determine the best model for predicting native species richness. The predicted native species richness from the best fit model was compared with the actual native species richness for each atoll in order to determine the residual values.

Residual values were then regressed again the human impact level in order to test the hypothesis that negative species richness residuals would be associated with greater human impact. This analysis was made at different scales, including the overall 111 atolls as well as subsets by archipelago ranging from 9 atolls (Cook) to 45 atolls (French Polynesia and Pitcairn). The mean level of human impact was also calculated for each atoll subset and compared to the *R* ^2^ goodness of fit of the biogeographic regression model obtained for each atoll subset. Finally, a focus with stepwise regression was also used to test the relationships between endemic species richness and the biogeographic variables for the overall 111 atolls as well as for French Polynesia and Pitcairn atolls (Marshall and Kiribati atolls lack endemics).

## 3 RESULTS

### 3.1 Composition of native species richness observed on the atolls

Native species richness mostly consisted of indigenous plants. Indigenous species richness found on the 111 atolls ranged from 3 (atoll of Johnston) to 176 (raised atoll of Niue) while endemic species (single‐atoll endemics plus archipelago‐level endemics) were found only on 24 atolls.

**Figure 2:**
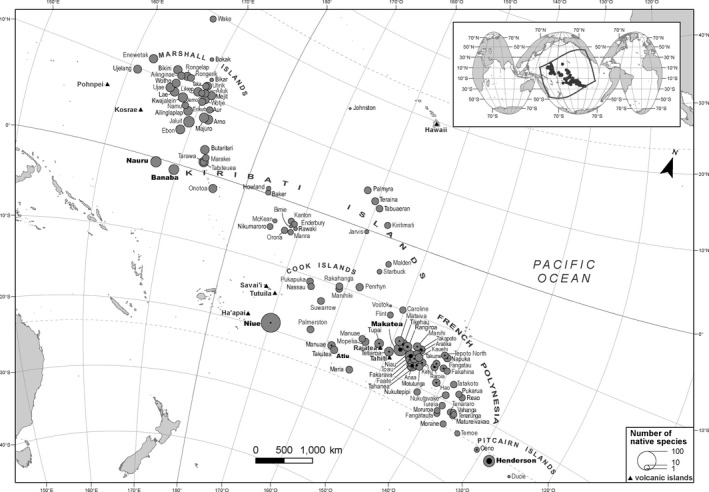
Distribution of native species richness (gray circle) including endemic species (black circle) on the 111 coral atolls surveyed (Pacific Ocean) with 106 low coral atolls and the nearest raised atolls ≥20 m a.s.l. (boldface). The nearest volcanic islands ≥100 km^2^ used in this study as stepping stone distance has been added (black triangle)

Thus, only 21.6% of the atolls sampled harbored endemic species (Figure 2). On these atolls, endemic species richness ranged from 1–10 species per atoll. A total of 26 endemics species was observed among which 69.2% were single‐atoll endemics (*n* = 18) and 30.8% were archipelago‐level endemics (*n* = 8) (Table 1). About 77.7% of the single‐atoll endemics were found on raised atolls ≥20 m a.s.l.. We found endemic species mainly on the raised atolls of Henderson (*n* = 10, Pitcairn atolls), Makatea (*n* = 6) and the slightly uplifted 5–6 m a.s.l. atolls of Anaa (*n* = 5) and Niau (*n* = 6) in French Polynesia. Only one endemic species was observed on Manuae (Cook atoll) and on the raised atoll of Niue (Table 1). Endemic species were not found on the Marshall and Kiribati atolls, nor on Nauru and Johnston (Figure 2).

**Table 1:** List of endemic species (including variety and subspecies levels) observed on the atolls sampled with “Single‐atoll endemics” (i.e., observed on one atoll) and “Archipelago‐level endemics” (i.e., observed on more than one atoll in the same archipelago)

Endemics species (*n* = 26)	Single‐atoll endemic	Archipelago‐level endemic	Atoll where the species is found	Archipelago
*Alyxia fosbergii* J.Florence [Apocynaceae]	+		Henderson	Pitcairn Islands
*Bidens hendersonensis* Sherff var. *hendersonensis* [Asteraceae]	+		Henderson	Pitcairn Islands
*Bidens hendersonensis* var. *oenoensis* Sherff [Asteraceae]	+		Oeno	Pitcairn Islands
*Geniostoma hendersonensis* H.St.John [Loganiaceae]	+		Henderson	Pitcairn Islands
*Homalium mouo* H.St.John [Salicaceae]	+		Makatea	Tuamotu (FP)
*Ixora fragrans* (Hook. & Arn.) A.Gray [Rubiaceae]	+		Henderson	Pitcairn Islands
*Ixora* sp. nov. [Rubiaceae]		+	Makatea, Niau, Anaa	Tuamotu (FP)
*Meryta* sp. nov. [Araliaceae]		+	Niau, Anaa	Tuamotu (FP)
*Myrsine hosakae* H.St.John [Primulaceae]	+		Henderson	Pitcairn Islands
*Myrsine niauensis* Fosberg and Sachet [Primulaceae]	+		Niau	Tuamotu (FP)
*Myrsine ovalis* var. *wilderi* Fosberg and Sachet [Primulaceae]	+		Makatea	Tuamotu (FP)
*Sideroxylon st‐johnianum* (H.J.Lam & B.Meeuse) Smedmark and Anderb. [Sapotaceae]	+		Henderson	Pitcairn Islands
*Pandanus* sp. nov. [Pandanaceae]	+		Makatea	Tuamotu (FP)
*Peperomia hendersonensis* Yunck. [Piperaceae]	+		Henderson	Pitcairn Islands
*Psychotria* sp. nov. [Rubiaceae]	+		Niau	Tuamotu (FP)
*Santalum insulare* var. *hendersonense* (Skottsb.) Fosberg and Sachet [Santalaceae]	+		Henderson	Pitcairn Islands
*Scrophulariaceae* sp. nov. [Scrophulariaceae]	+		Makatea	Tuamotu (FP)
*Sesbania coccinea* subsp. *atollensis* var. *atollensis* (H.St.John) Sachet [Fabaceae]		+	Anaa, Niau, numerous atolls	Tuamotu (FP)
*Sesbania coccinea* subsp. *atollensis* var. *parkinsonii* Sachet [Fabaceae]		+	Tupai, Tetiaroa	Société (FP)
*Sesbania coccinea* subsp. *atollensis* var. *tuamotensis* (F.Br.) Sachet [Fabaceae]		+	Anaa, numerous atolls	Tuamotu (FP)
*Xylosma suaveolens* subsp. *haroldii* Sleumer [Salicaceae]	+		Henderson	Pitcairn Islands
*Glochidion pitcairnense* (F.Br.) H.St.John [Phyllanthaceae]		+	Henderson	Pitcairn Islands
*Glochidion tuamotuense* J. Florence [Phyllanthaceae]	+		Niau	Tuamotu (FP)
*Glochidion wilderi* J. Florence [Phyllanthaceae]		+	Makatea, Anaa	Tuamotu (FP)
*Microsorum katuii* (Brownlie) Sykes [Polypodiaceae]		+	Manuae	Cook Islands
*Peperomia pallida* var. *niueana* Yunck. [Piperaceae]	+		Niue	Niue

Note

FP: French Polynesia.

### 3.2 Native species richness in relation to the biogeographic variables

On the 111 coral atolls surveyed, native species richness was positively related to atoll area and highest atoll elevation. Native species richness was negatively related with the distance from (a) the nearest raised atoll ≥20 m and (b) from the nearest volcanic island ≥100 km^2^ (Table 2). This confirms that native species richness decreases in the atolls with increasing distance to both these islands types. Significant predictors changed with the different archipelago and atoll subsets sampled. However, the regression model identified significant predictors to explain native species richness for each atoll subset, ranging from *R* ^2^ **= 0.436, *p* ≤ 0.01 (Kiribati atolls) to *R* ^2^ **= 0.877, *p* ≤ 0.01 (Cook atolls) (Table 2). When added to the biogeographic models, level of human impact did not contribute significantly to native species richness (Table 2).

**Table 2:** Native species richness versus the five biogeographic variables and human impact tested with stepwise regression model on 111 atolls (Pacific Ocean). Detail is given per archipelago. The atolls of Niue, Nauru, and Johnston (USA) were included in the 111 atolls

Biogeographic variables	Value	*SD*	*t*	Pr>|*t*|	Lower bound (95%)	Upper bound (95%)	Adjusted *R* ^2^
All atolls (*n* = 111)							
1. Coastline length (km) L							0.645
2. Atoll area (km^2^)	0.40	0.06	6.77	<0.0001	0.28	0.52	
3. Highest elevation (m)	0.39	0.06	6.40	<0.0001	0.27	0.52	
4. Distance from the nearest raised atoll (km)	−0.21	0.07	−3.18	0.002	−0.34	−0.08	
5. Distance from the nearest volcanic island (km)	−0.29	0.06	−4.63	<0.0001	−0.42	−0.17	
Level of human impact				*ns*			
Marshall atolls (*n* = 28)							
1. Coastline length (km) L							0.673
2. Atoll area (km^2^)	0.41	0.13	3.13	0.005	0.14	0.67	
3. Highest elevation (m)	0.35	0.13	2.61	0.015	0.07	0.63	
4. Distance from the nearest raised atoll (km)				*ns*			
5. Distance from the nearest volcanic island (km)	−0.41	0.11	−3.60	0.001	−0.65	−0.18	
Level of human impact				*ns*			
Kiribati atolls (*n* = 26)							
1. Coastline length (km)	0.52	0.15	3.39	0.003	0.20	0.84	0.436
2. Atoll area (km^2^) L							
3. Highest elevation (m)	0.57	0.15	3.73	0.001	0.25	0.89	
4. Distance from the nearest raised atoll (km)				*ns*			
5. Distance from the nearest volcanic island (km)				*ns*			
Level of human impact				*ns*			
Cook atolls (*n* = 9)							
1. Coastline length (km) L							0.877
2. Atoll area (km^2^)	0.86	0.13	6.68	0.001	0.55	1.18	
3. Highest elevation (m)				*ns*			
4. Distance from the nearest raised atoll (km)	−0.70	0.13	−5.45	0.002	−1.02	−0.39	
5. Distance from the nearest volcanic island (km)				*ns*			
Level of human impact				*ns*			
French Polynesia and Pitcairn atolls (*n* = 45)							
1. Coastline length (km) L							0.770
2. Atoll area (km^2^)	0.31	0.08	3.74	0.001	0.14	0.49	
3. Highest elevation (m)	0.45	0.08	5.88	<0.0001	0.30	0.61	
4. Distance from the nearest raised atoll (km)	−0.25	0.09	−2.80	0.008	−0.43	−0.07	
5. Distance from the nearest volcanic island (km)	−0.27	0.08	−3.39	0.002	−0.43	−0.11	
Level of human impact				*ns*			

Note

*ns*: not significant (*p*‐value to enter <0.01; *p*‐value to remove > 0.10); L: left out from regression.

In the 111 atolls sampled, we observed a positive relationship between endemic species richness and highest atoll elevation, and a negative relationship with the distance from the nearest raised atoll ≥20 m a.s.l. (Table 3). The overall adjusted *R* ^2^ was low with a value of 0.218 (Table 3). On the atolls of French Polynesia and Pitcairn Islands, endemic species richness was positively related to highest atoll elevation, and negatively related to distance from the nearest raised atoll ≥20 m a.s.l., with an adjusted *R* ^2^ of 0.458, *p* ≤ 0.01 (Table 3).

**Table 3:** Endemic species richness (single‐atoll endemics plus archipelago‐level endemics) in relation to the five biogeographic variables and human impact on the 111 atolls surveyed with a focus on the atolls of French Polynesia and Pitcairn Islands (stepwise regression model)

Biogeographic variables	Value	*SD*	*t*	Pr>|*t*|	Lower bound (95%)	Upper bound (95%)	Adjusted *R* ^2^
All atolls (*n* = 111)							
1. Coastline length (km) L							0.218
2. Atoll area (km^2^)				*ns*			
3. Highest elevation (m)	0.28	0.09	3.16	0.002	0.10	0.45	
4. Distance from the nearest raised atoll (km)	−0.32	0.09	−3.69	0.000	−0.50	−0.15	
5. Distance from the nearest volcanic island (km)				*ns*			
Level of human impact				*ns*			
French Polynesia and Pitcairn atolls (*n* = 45)							
1. Coastline length (km) L							0.458
2. Atoll area (km^2^)				*ns*			
3. Highest elevation (m)	0.43	0.12	3.66	0.001	0.19	0.67	
4. Distance from the nearest raised atoll (km)	−0.42	0.12	−3.60	0.001	−0.66	−0.19	
5. Distance from the nearest volcanic island (km)				*ns*			
Level of human impact				*ns*			

Note

*ns*: not significant (*p*‐value to enter <0.01; *p*‐value to remove > 0.10); L: left out from regression.

### 3.3 Model residuals versus level of human impact

**Figure 3:**
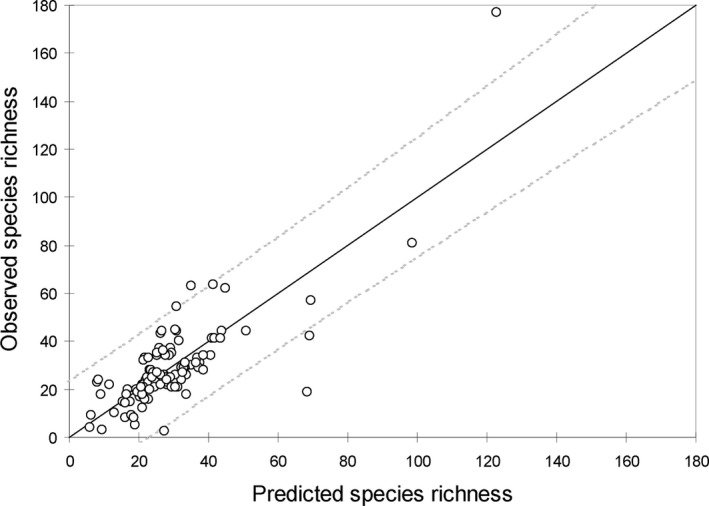
Observed (circle) versus predicted (continuous line) native species richness based on the stepwise regression model—native species richness relationship across the 111 atolls surveyed (Pacific Ocean). Interval between dashed gray lines shows 95% of the residuals

We found two positive and three negative residual outliers (Figure 3). The five atolls identified as significant residual model outliers are given in Table 4. Residuals of raised atolls of Henderson (Pitcairn) and Niue, both of which had low human impact indices, were significantly above the expected species richness value. In contrast, the atoll of Vostok, the raised atoll of Banaba and the atoll of Kiritimati (Kiribati) were below the expected richness value. The level of human impact was high on these two last atolls (Table 4).

**Table 4:** The five atolls identified as significant residual model outliers (Figure 3) in the 111 atolls sampled (Pacific Ocean). Some special outlier characteristics with the level of human impact have been added

Residuals	Special physical characteristics	Other special features	Level of human impact
Positive outliers			
Niue	Raised atoll (73 m a.s.l., second biggest atoll sampled)	Topographic variability; protected forest and sacred forest (called *Tapu* area)	0
Henderson (Pit.)	Raised atoll (33 m a.s.l.)	Uninhabited, endemic birds	0
Negative outliers			
Vostok (K)	Low atoll (smallest atoll sampled, 0.02 km^2^)	Formerly invaded by rats; closed forest of *Pisonia grandis*; no freshwater lens	3
Banaba (K)	Raised atoll (81 m a.s.l.)	Military installation; devastation of World War II; phosphate mining over 80 years	6
Kiritimati (K)	Low atoll (biggest atoll sampled, 388.4 km^2^)	Military installation; devastation of World War II; phosphate mining; nuclear testing	10

Note

K: Kiribati atolls; Pit.: Pitcairn atolls.

**Figure 4:**
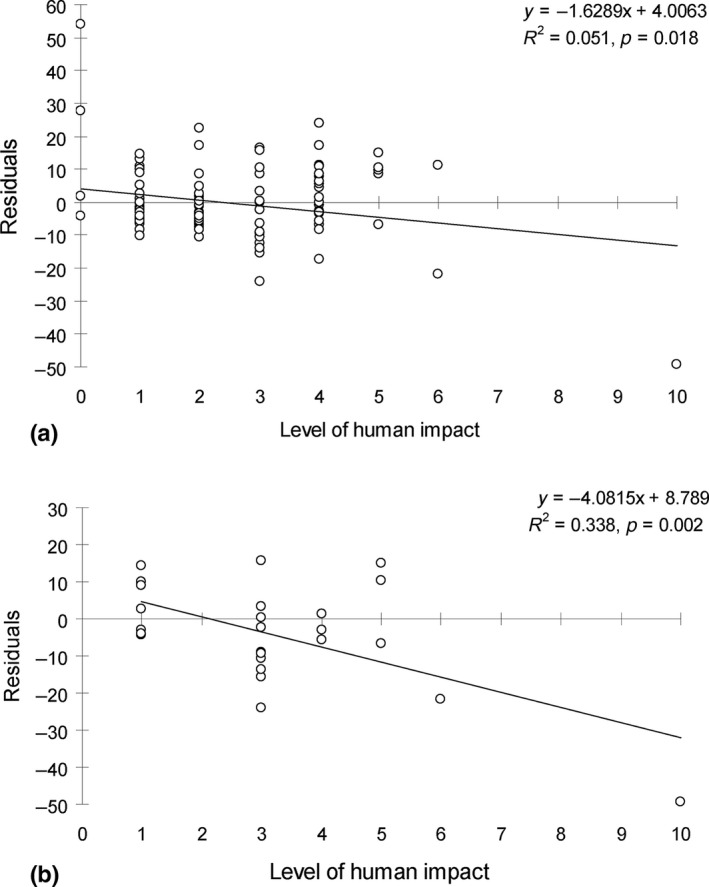
(a–b) Residuals in regression model versus the level of human impact in (a) the 111 atolls and (b) the subset of Kiribati atolls (Pacific Ocean)

**Figure 5:**
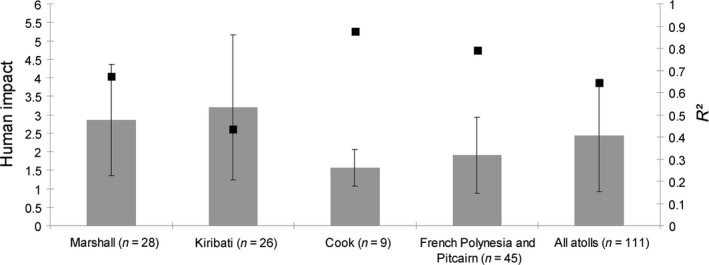
Mean level of human impact (gray bars) compared with adjusted *R* ^2^ regression model—species richness relationship (black squares) per atoll subsets (Pacific Ocean). Error bars refer to standard deviation

Residuals in the regression model—species richness relationship were barely related to human impact across all 111 atolls (*R* ^2^ = 0.051, *p* = 0.018; Figure 4a) but were significantly related to human impact for the Kiribati atolls (*R* ^2^ = 0.338, *p* = 0.002; Figure 4b). The level of human impact was stronger on the atolls of Kiribati and Marshall than those of Cook, French Polynesia and Pitcairn, and these island groups had lower *R* ^2^ values for models using biogeographic variables (Figure 5).

## 4 DISCUSSION

### 4.1 How do the findings fit with the general dynamic model?

Biogeographic variables were significant predictors of native species richness for every atoll subset tested, explaining between 43.6% and 87.7% of the native species richness variation on the atolls. These findings suggest that native species richness on coral atolls is mainly controlled by physical characteristics and processes. This result fits the final stage of the GDM where the realized species richness (R) is expected to decline with the decreasing potential carrying capacity (K) (here, affected by elevation and atoll area). At this final stage of the GDM, the speciation rate (S) is close to zero, while the extinction rate of endemic species (E) increases; in this study, 78.3% of the atolls sampled harbored no endemic species.

### 4.2 Native species richness

Overall, native species richness (indigenous plus endemic) on the 111 atolls was significantly related to highest atoll elevation supporting the hypothesis that tidal waves and cyclones may be important factors controlling, at least partially, indigenous, and endemic plant communities on the coral atolls. Some authors have reported low native plant diversity on the low atolls due to frequent marine submersion during cyclones (Bayliss‐Smith, 1988; Fosberg, 1991; Waldren et al., 1995; Woodroffe & Stoddard, 1992) and these events are episodic in the Pacific Islands (de Scally, 2008; Goff et al., 2011; Larrue, 2014; Larrue & Chiron, 2010).

The distance from a high volcanic island and the distance from a raised atoll were important to explain native species richness pattern across the 111 remote atolls. This illustrates that stepping stone distances are important in increasing the explanatory power of isolation (i.e., the distance from the species pool) (Rosindell & Phillimore, 2011; Weigelt & Kreft, 2013).

Finally, native species richness on the 111 atolls was significantly related to atoll area. First, species–area relationships (SAR) of atolls may be related to some habitat diversity. Indeed, despite homogenous geomorphology and landform, large atolls sometimes harbor a swampy habitat in the middle of the islet. The influence of the salt and the wind could be variable on large atolls providing more diverse ecological conditions. Second, large atolls may receive more material and resources deposited by the ocean currents, increasing species richness on the “subsidized islands” (see the “subsidized island” biogeography hypothesis (Anderson & Wait, 2001; Barrett, Wait, & Anderson, 2003)). Third, seeds dispersion of indigenous plants on the Pacific atolls often occurs from ocean dispersal and birds zoochory, most are readily bird or ocean current dispersed (e.g., Florence et al., 1995; Mueller‐Dombois, 2002). Consequently, large atolls may experience greater visitation by birds, increasing chances that birds may rescue plant species via interatoll movement. Other atoll variables are also related to atoll area and could affect native species richness. It is well known that freshwater lens is an important predictor for species richness and this lens is more developed on large atolls (Whitehead & Jones, 1969). Although this information is poorly documented on the atolls sampled, some atolls are too small to support a freshwater lens and it is notably the case for Vostok Atoll (Kiribati) which has very restricted species richness, consequently.

In this study, significant predictors sometimes changed when different archipelago and atoll subsets were analyzed. Subset sampling reduces overall statistical power, so fewer significant variables may be expected when analyzing subsets. In addition, different significant predictors can be also explained by some idiosyncratic phenomena (e.g., Triantis et al., 2006), probably including the level of human impacts in the archipelago.

### 4.3 Endemic species richness and biogeographic variables

Whereas endemic species were poorly represented on the 111 atolls, a significant relationship was observed with (a) highest atoll elevation and (b) the distance from the nearest raised atolls. Typically, species richness of atolls sampled is composed of widespread easily dispersed native species, with a low number of endemics probably due to recent emergence of most atolls. Indeed, around 8,500 to 4,000 year BP the Holocene sea level was 1.0–2.6 m above modern sea level (Dickinson, 2004). The low atolls were flooded and the Pleistocene platform would have been completely or partially submerged (Ohde et al., 2002; Szabo et al., 1985), depending on atoll elevation. The Holocene highstand had begun to decline by ~3,000 year BP in the Pacific Islands, but some geographic variation of sea‐level history has been identified (Lambeck, 2002) and the Holocene highstand persisted until ~2,000 year BP in the central Pacific atolls (Dickinson, 2003; Pirazzoli & Montaggioni, 1988). Thus, most low atolls have only re‐emerged around 2,000–1,500 year BP, except for the raised atolls sampled which remained emergent over the Pleistocene and Holocene period.

Our findings support the idea that after eustatic sea‐level variation and the Holocene highstand, endemic plants (here, archipelago‐level endemics) have colonized some re‐emerged atolls from the nearest raised atoll. Archipelago‐level endemics then were able to maintain persistent populations only on the highest atolls, that is, above the last Holocene highstand and protected from marine submersion during cyclonic swells and episodic contemporary instability.

In addition, single‐atoll endemics were mostly observed on the raised atoll and this result was congruent with the hypothesis that they are refugia for vascular plants. However, some single‐atoll endemics were also observed on the atoll of Niau (6 m a.s.l.) and Anaa (5 m a.s.l.). In fact, a part of Pleistocene limestone is exposed at the surface of Niau and Anaa (Pirazzoli, Koba, Montaggioni, & Person, 1988) suggesting that these slightly uplifted atolls were not entirely submerged during the last interglacial period (lack of Holocene sea‐level deposit).

### 4.4 Influence of human impacts

An influence of human impact was detected with residuals in the biogeographic regression model, supporting the prediction of decreasing native species richness due to human impact. In addition, the *R* ^2^ of the regression model testing effects of biogeographic variables on native species richness relationship on the Marshall and Kiribati atolls were lower than those of Cook or French Polynesia and Pitcairn atolls. This finding might be related to anthropogenic disturbances. Indeed, the level of human impact was high on the Marshall and Kiribati atolls compared to Cook, French Polynesia and Pitcairn atolls. For example, the raised atoll of Banaba (Kiribati) has been exploited for open‐cast phosphate mining over 80 years (Thaman & Samuelu, 2016; Thaman, Fosberg, Manner, & Hassall, 1994) and the model residual of Banaba was significantly below the expected value from the biogeographic regression model. Additionally, it is well known that some Kiribati and Marshall atolls were devastated during the World War II and during nuclear tests in the 1950s–1960s (e.g., Bordner et al., 2016; Fosberg, 1953, 1985; Richards Zoe, Beger, Pinca, & Wallace, 2008). Accordingly, the residual species richness of Kiritimati Atoll (Kiribati) was significantly lower than expected. In addition to human settlement, coconut monoculture and destruction during World War II, this atoll has experienced 24 nuclear tests between 1957 and 1962 (Robbins, 1991). So, human disturbances on the Marshall and Kiribati atolls might explain the lower fit of the biogeographic regression model there in comparison with the Cook or French Polynesia and Pitcairn atolls, which have been historically less disturbed by human activities.

## 5 CONCLUSION

Native species richness on Pacific atolls can be predicted by a combination of some simple atoll characteristics and distances. Here, a stepwise regression model was a suitable linear model to explain native plant species richness across all atolls sampled, with only a few notable outliers being detected. So, the capacity of the atolls to “capture species” and maintain native plants strongly relates to a combination of simple spatial and abiotic predictors. The findings fit with the final stage of general dynamic model, which predicts that native species richness before island submergence is mainly controlled by physical characteristics and processes. However, different levels of anthropogenic disturbance seem to have altered the predicted pattern of native species richness, which may explain lower model fit in some atoll subsets. The findings suggest that effects of human impact should be further investigated for a better understanding of biodiversity patterns in island biogeography.

## CONFLICT OF INTEREST

None Declared.

## AUTHOR CONTRIBUTIONS

S.L. and J.‐F. B. conceived the ideas; S.L., J.‐F. B. and J.C. collected botanical and geographical data; S.L., C.D., S.B., and R.O. analyzed the data; and S.L. and C.D. led the writing. J.C. realized the Figure 2.

## DATA ACCESSIBILITY

Data are publicly available from the Dryad Digital Repository at https://doi.org/10.5061/dryad.1v2k8b1
